# Data on HepG2 cells changes following exposure to cadmium sulphide quantum dots (CdS QDs)

**DOI:** 10.1016/j.dib.2016.12.051

**Published:** 2016-12-31

**Authors:** Laura Paesano, Alessio Perotti, Annamaria Buschini, Cecilia Carubbi, Marta Marmiroli, Elena Maestri, Salvatore Iannotta, Nelson Marmiroli

**Affiliations:** a Department of Life Sciences, University of Parma, Parco Area delle Scienze 11/A, Parma 43124, Italy; b Department of Biomedical, Biotechnological and Traslational Sciences (S.Bi.Bi.T), University of Parma, Via Gramsci 14, Parma 43126, Italy; c Istituto dei Materiali per l׳Elettronica ed il Magnetismo (IMEM-CNR), Parco Area delle Scienze 37/A, Parma 43124, Italy

## Abstract

The data included in this paper are associated with the research article entitled "Markers for toxicity to HepG2 exposed to cadmium sulphide quantum dots; damage to mitochondria" (Paesano et al.) [1]. The article concerns the cytotoxic and genotoxic effects of CdS QDs in HepG2 cells and the mechanisms involved. In this dataset, changes in expression levels of candidate genes are reported, together with details concerning synthesis and properties of CdS QDs, additional information obtained through literature survey, measures of the mitochondrial membrane potential and the glutathione redox state.

**Specifications Table**

**Table:** 

Subject area	*Biology*
More specific subject area	*Toxicogenomics, transcriptomics*
Type of data	*Table, image (x-ray, microscopy), text file, graph, figure*
How data was acquired	*Electron microscopy (JEM-2200 FS transmission electron microscope, ESEM Quanta 250FEG)*
	*MTS assay (CellTiter 96*^®^*AQ*_*ueous*_*One Solution Cell Proliferation Assay)*
	*Real Time PCR and hierarchical clustering (TaqMan Custom Array Plates, Applied Biosystems 7900HT Fast Real-Time PCR System),*
	*DTNB assay [5,5′-dithiobis(2-nitrobenzoic acid)]*
	*Flow cytometry (FC500 flow cytometer)*
Data format	*Filtered and analyzed*
Experimental factors	*HepG2 cells were exposed to a toxic acute dose of CdS QDs corresponding to the IC*_*50*_*, and to two sub-toxic doses for different time periods*
Experimental features	*Cells were cultured in Dulbecco׳s Modified Eagle׳s Medium (DMEM) containing 10% (v/v) fetal bovine serum (FBS)*, 50 U mL^−1^*penicillin/streptomycin and 1% (w/v) L-glutamine in an incubator at* 37 °C *and 5% CO*_*2*_
Data source location	*Parma, Italy*
Data accessibility	*Data are available within this article*

**Value of the data**

• The dataset provides a list of candidate genes useful for comparing effects of nanomaterials in other cell systems.
• The data may be useful for other researchers analysing mitochondrial dysfunctionalities in stressed cells.
• The literature survey may be useful for planning additional experiments on risk assessment of cadmium-based quantum dots.

## 1 Data

Table 1 shows a survey of the most recent literature on experimentation with animal cell lines and cadmium-based quantum dots. It details the main parameters which can be assessed to estimate the functionality and viability of cells and describes the main changes detected in cells. Particular reference has been made to instances of oxidative stress, apoptosis and autophagy. In addition Table 2 reports a comparison of the effects of exposure to CdS QDs and Cd^2+^ ions. Table 3 reports in details the experimental conditions chosen for treating HepG2 cells with CdS QDs. Table 4 lists the changes in gene expression of candidate genes upon exposure to toxic and subtoxic doses of CdS QDs. Genes are grouped according to their involvement in relevant cellular processes. Table 5 lists the changes in gene expression determined after quantitative reverse transcriptase PCR (qRT-PCR) for specific genes chosen on the basis of their involvement in relevant cellular processes.

## 2 Experimental design, materials and methods

### 2.1 Synthesis and characterization of CdS QDs

**Fig. 1:**
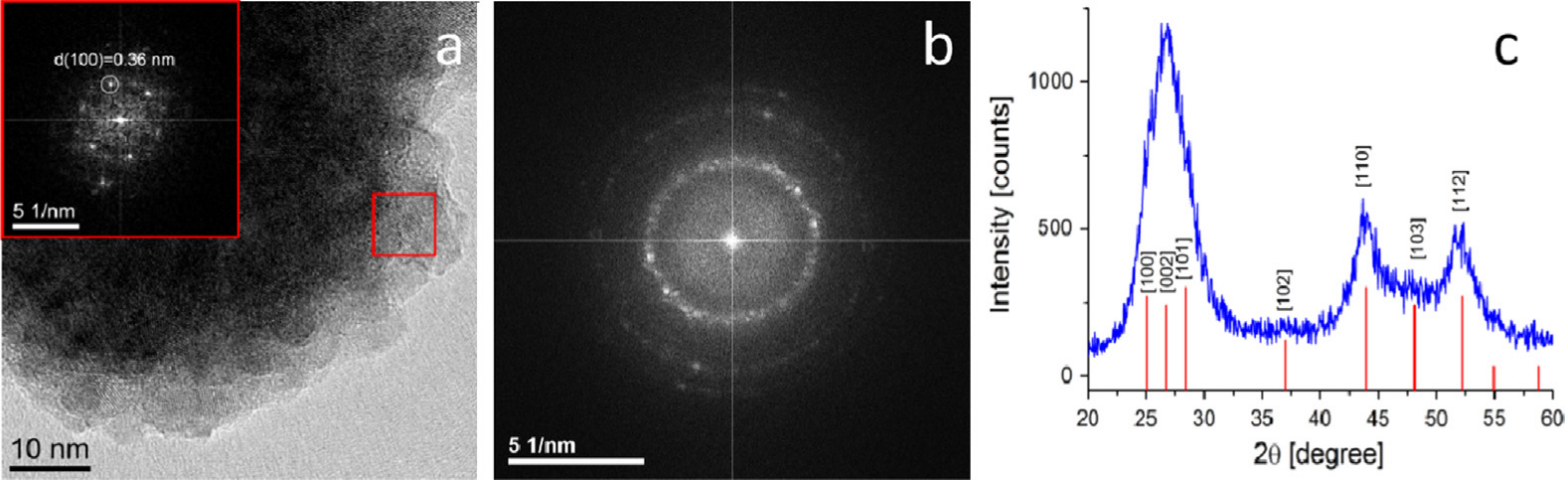
HRTEM image of ligand-free QDs assembly. (a) A CdS QDs aggregate after solvent evaporation. (b) Fourier transform analysis of the whole HRTEM image. (c) X-ray diffraction pattern.

**Fig. 2:**
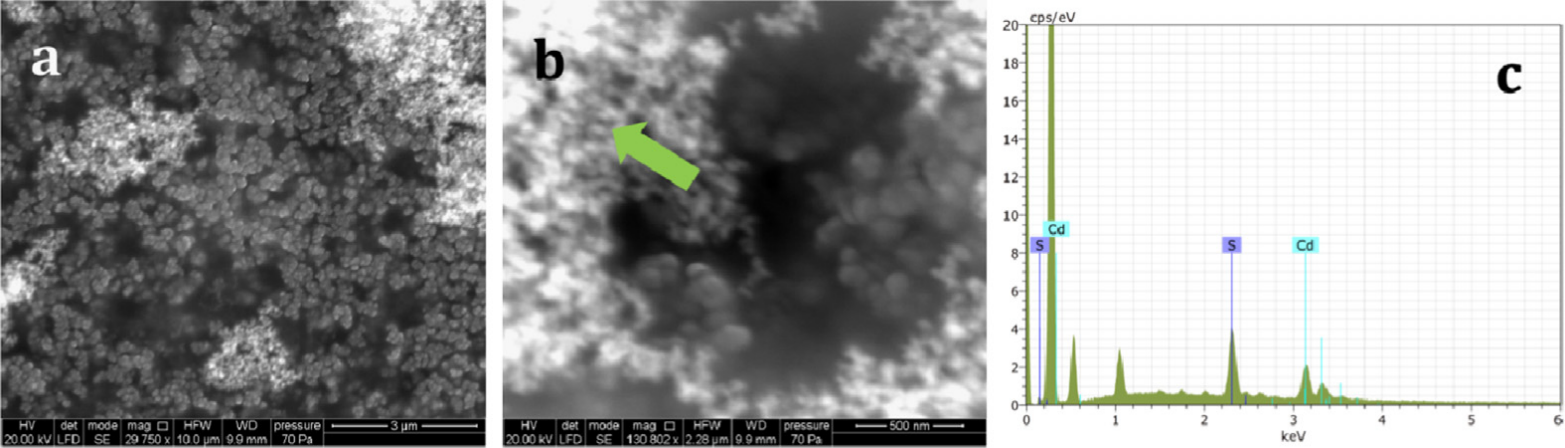
ESEM/EDX image of a 80 mg L^−1^ ligand-free suspension of CdS QD. The panels in (a) and (b) show SEM images at different magnifications. The green arrow in (b) indicates the point where EDX was performed. (c) EDX spectra of CdS QDs, reporting X-ray emission lines for Cd and S.

The method^1^ used to synthesize CdS QDs followed Villani et al. [28], and the synthesis was performed by IMEM-CNR (Parma, Italy). X-ray diffraction (XRD) was carried out using an ARL-X’Tra device (Thermo Fisher Scientific, Waltham, MA, USA). A field emission high resolution (Scherzer resolution of ~0.19 nm) JEM-2200 FS transmission electron microscope (JEOL Ltd., Tokyo, Japan) operating at 200 kV, was used to examine QD structure. The aggregation of a group of QDs following solvent evaporation (Fig. 1a) was due to the lack of capping molecules at the QD surface. The corresponding reduced Fourier transform (FT) in the inset confirms the hexagonal structure (greenockite, P63mc) of as-synthesized CdS QDs (*d*=0.36 nm in agreement with standard card JCPDS no. 80-0006). The FT of the whole high resolution transmission electron microscope (HRTEM) image is presented in Fig. 1b. The expected ring feature arising from the random orientation of CdS crystals is clear, as is the overlap of (100), (002) and (101) reflections of the wurtzite structure (at high d values) due to low dimension peak broadening. Such features are in agreement with the XRD pattern shown in Fig. 1c. All peaks have been indexed according to the structure of greenockite and no other reflections arising from possible impurities are observed. A Scherrer calculation based on the FWHM (full width at half maximum) of the three main peaks produced an estimated mean size of ~6 nm. An ESEM (environmental scanning electron microscopy) Quanta 250FEG (FEI Co., Hillsboro, OR, USA) together with a QUANTAX EDS (energy-dispersive systems) XFlash® 6T detector series and the ESPRIT 2 analytical methods interface (Bruker, Berlin, Germany) was used to determine CdS QDs morphology and elemental content. Single 1 mL drops containing 80 mg L^−1^ CdS QDs were left to dry on an scanning electron microscopy (SEM) stub covered with carbon tape in a protected environment. Seven stubs were analysed during one round of experiments. The working parameters for SEM imaging and X-ray spectra acquisition were: pressure: 70 Pa, working distance: 9.9 mm, acceleration voltage: 20 KeV. SEM images of a CdS QDs drop at 29,750x magnification (Fig. 2a) and at 130,802x magnification (Fig. 2b) show the nanocrystals are grouped into small agglomerates of 50–100 nm. The energy-dispersive X-ray analysis (EDX) (Fig. 2c) of the point indicated by the green arrow in Fig. 2b reveals clear emission lines for Cd Lα1 and Lβ1 at 3.133 and 3.316 eV. The S Kα1 and Kβ1 lines at 2.308 and 2.464 eV are also visible.

### 2.2 Cell culture

**Fig. 3:**
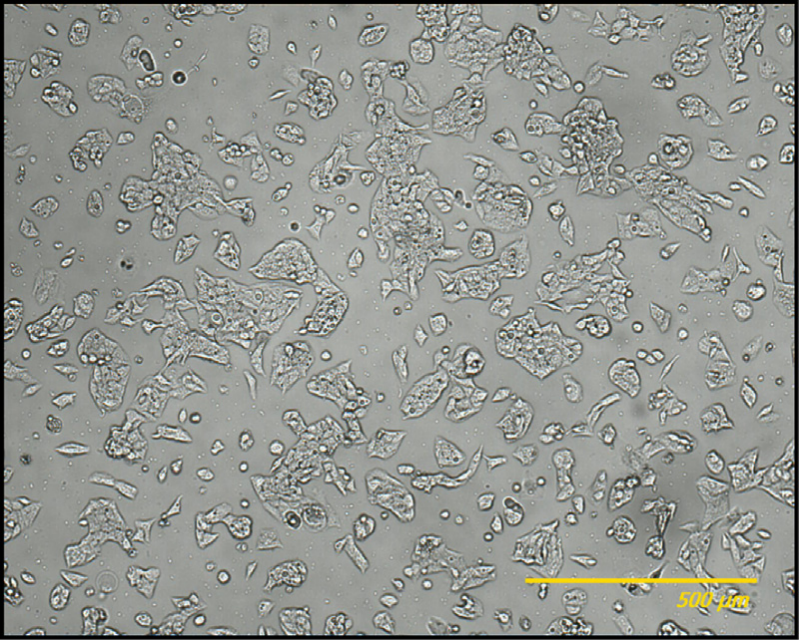
The appearance of the cultured HepG2 cells. The bar corresponds to 500 µm.

HepG2 cells (Fig. 3) were cultured in Dulbecco׳s Modified Eagle׳s Medium (DMEM) contained 10% (v/v) fetal bovine serum (FBS), 50 U mL^−1^ penicillin/streptomycin and 1% (w/v) L-glutamine in an incubator at 37 °C and 5% CO_2_. Cells were exposed to different doses of CdS QDs.

### 2.3 Cytotoxicity assay

**Fig. 4:**
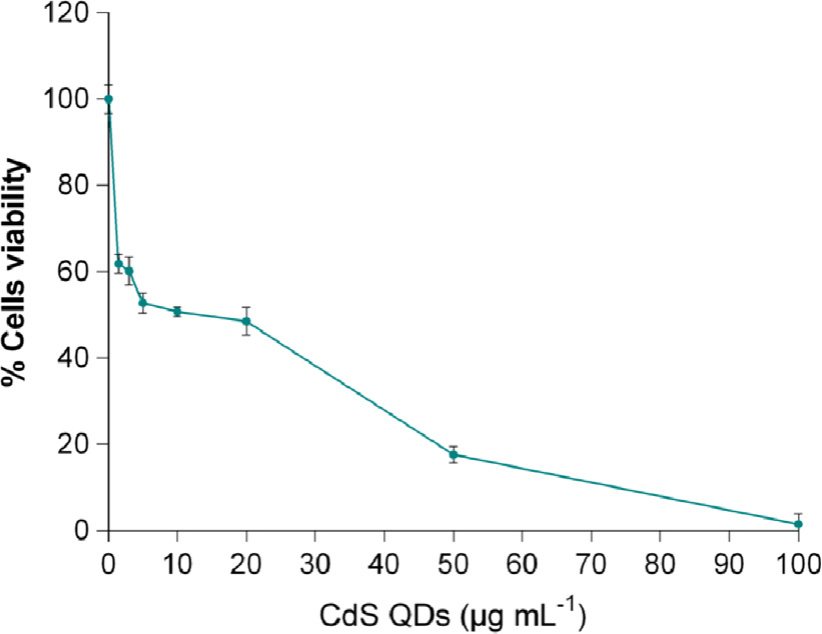
Cytotoxicity of CdS QDs on HepG2 cells. The inhibitory effect of various doses (0.5–100 µg mL^−1^) on cell viability was determined by MTS assay. The percentage reduction in viability was calculated from absorbance measurements of treated and untreated cell cultures. Data showed in figure are representative of three independent experiments, performed in triplicate.

The cytotoxicity of the CdS QDs (Fig. 4) was evaluated by CellTiter 96^®^ AQ_ueous_ One Solution Cell Proliferation Assay (Promega, Madison, WI, USA) according to the manufacturer׳s protocol. In this assay, cell viability was assumed to be proportional to the quantity of formazan generated by the reduction of MTS. Data obtained were also used in Paesano et al. [1].

### 2.4 Detection of glutathione

**Fig. 5:**
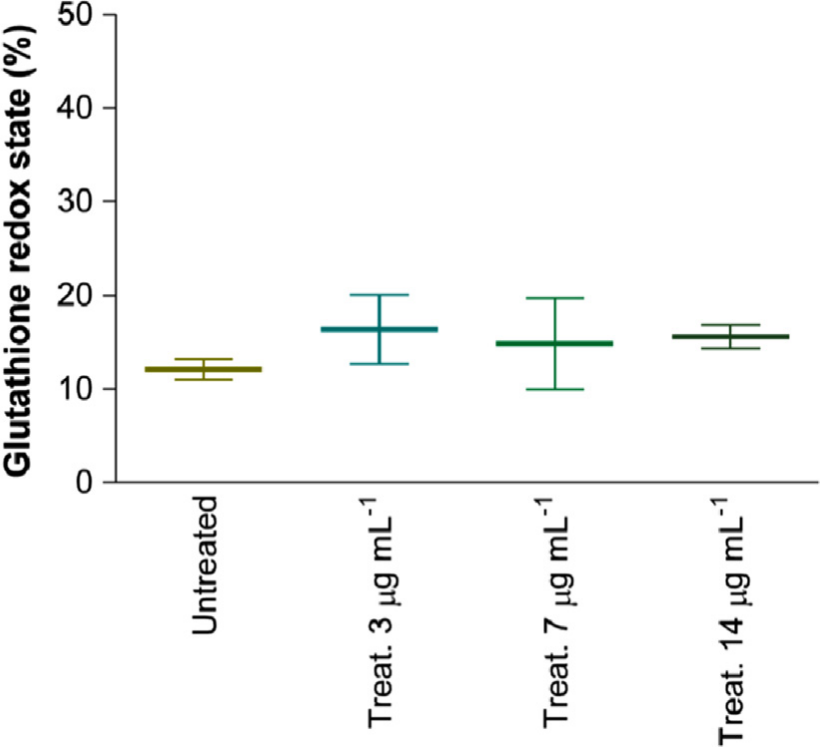
Effect of CdS QDs on the glutathione redox state (GSH/GSSG) of HepG2 cells. Cells were exposed to either 3, 7 or 14 µg mL^−1^ CdS QDs for 24 h. Untreated cells cultured were run in parallel. Aflatoxin B treatment was used as positive control. Data showed in figure are representative of three independent experiments, performed in triplicate.

The cellular level of glutathione (GSH) was determined by an assay based on the conversion of 5,5′-dithiobis(2-nitrobenzoic acid) (DTNB) (Sigma-Aldrich) to the yellow-coloured product 2-nitro-5-thiobenzoic acid (TNB) (Fig. 5).

### 2.5 Assay on mitochondrial integrity

**Fig. 6:**
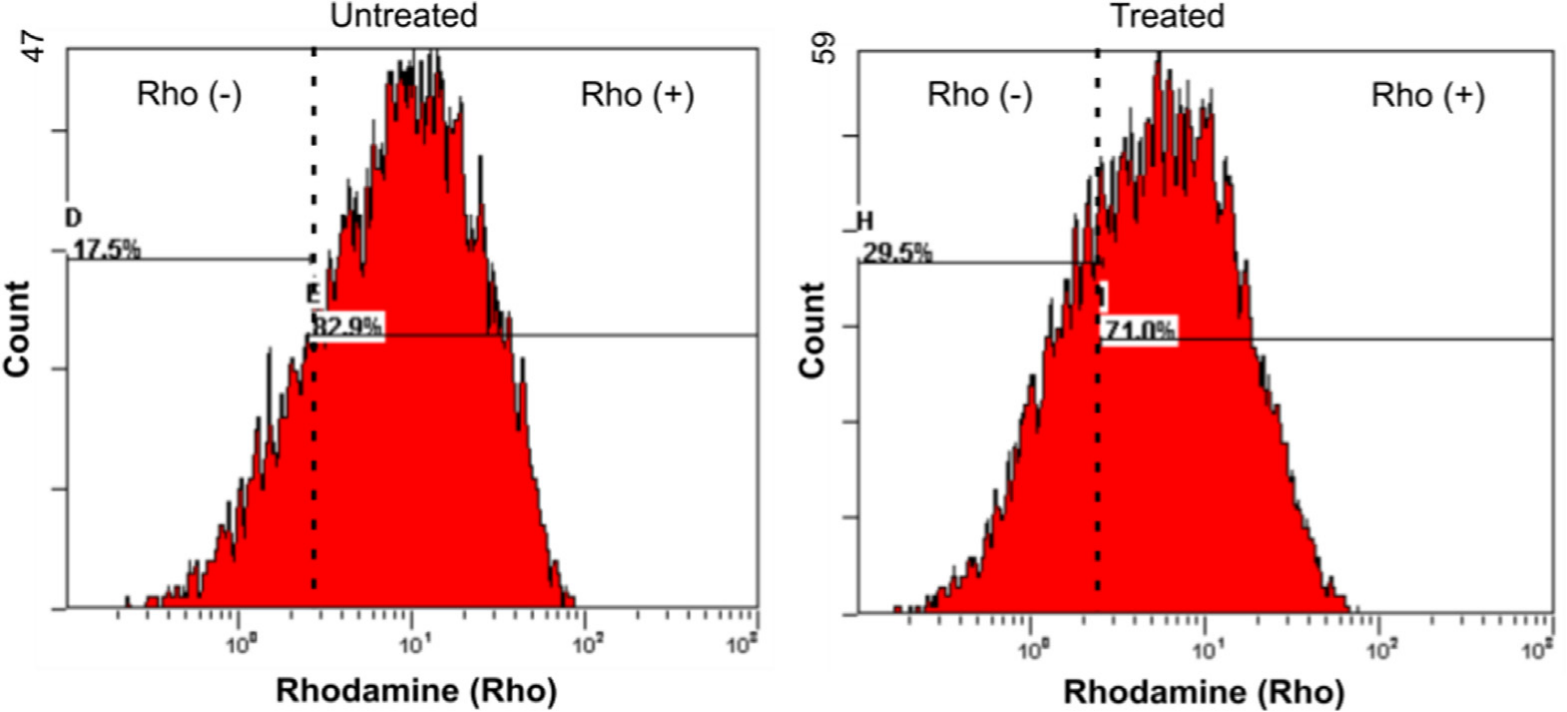
Effect of CdS QDs treatment on the mitochondrial membrane potential, as quantified by flow cytometry of rhodamine-stained cells. Cell were exposed to 14 µg mL^−1^ CdS QDs for 4 h. Rhodamine unstained [Rho(−)] and stained cells [Rho(+)] were gated and their fluorescence signal was quantified. Comparison of the two groups indicated a 12% reduction in fluorescence between the treated and untreated cells. The data shown in figure are representative of three independent experiments, performed in triplicate.

HepG2 cells were exposed to a range of CdS QDs concentrations and were subsequently stained for 30 min in 40 nM rhodamine B hexyl ester (ThermoFisher Scientific, Waltham, MA, USA), rinsed in PBS, harvested by centrifugation (800 g, 5 min) and re-suspended in PBS with 1% (v/v) FBS. The suspension was analyzed using a FC500 flow cytometer (Beckman Coulter Inc.) (Fig. 6).

### 2.6 Analysis of transcriptomic data

**Fig. 7:**
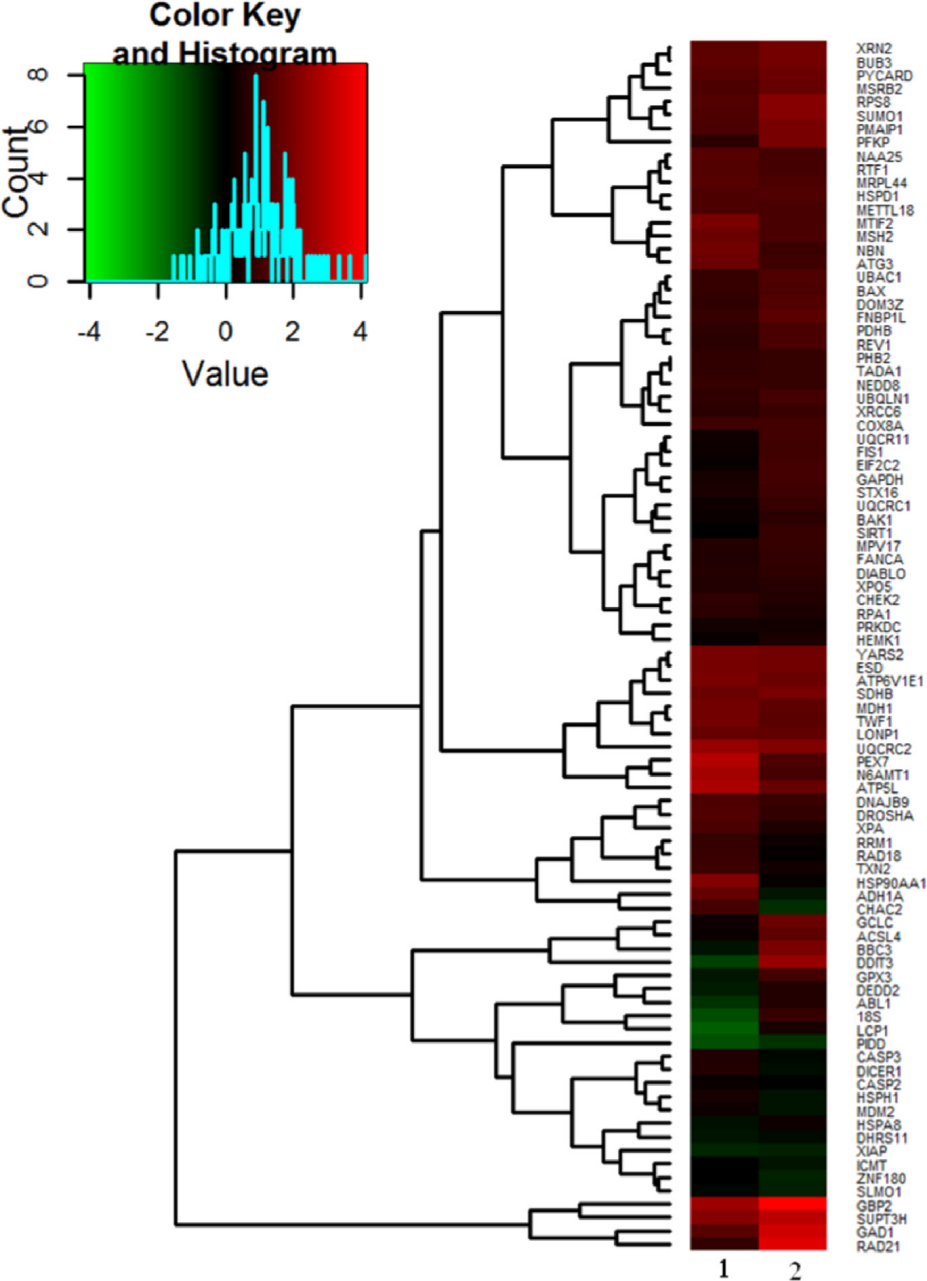
Hierarchical clustering (heatmap) of relative transcript abundances obtained by TaqMan^®^ Custom Array Plates in qRT-PCR. Up-regulated genes are shown in red and down- regulated ones in green. The colour intensity reflects the transcript׳s abundance. HepG2 cells were exposed for 24 h to either (1) 3 or (2) 14 µg mL^−1^ CdS QDs. Data showed in figure are representative of three independent experiments, each performed in triplicate for each condition.

**Fig. 8:**
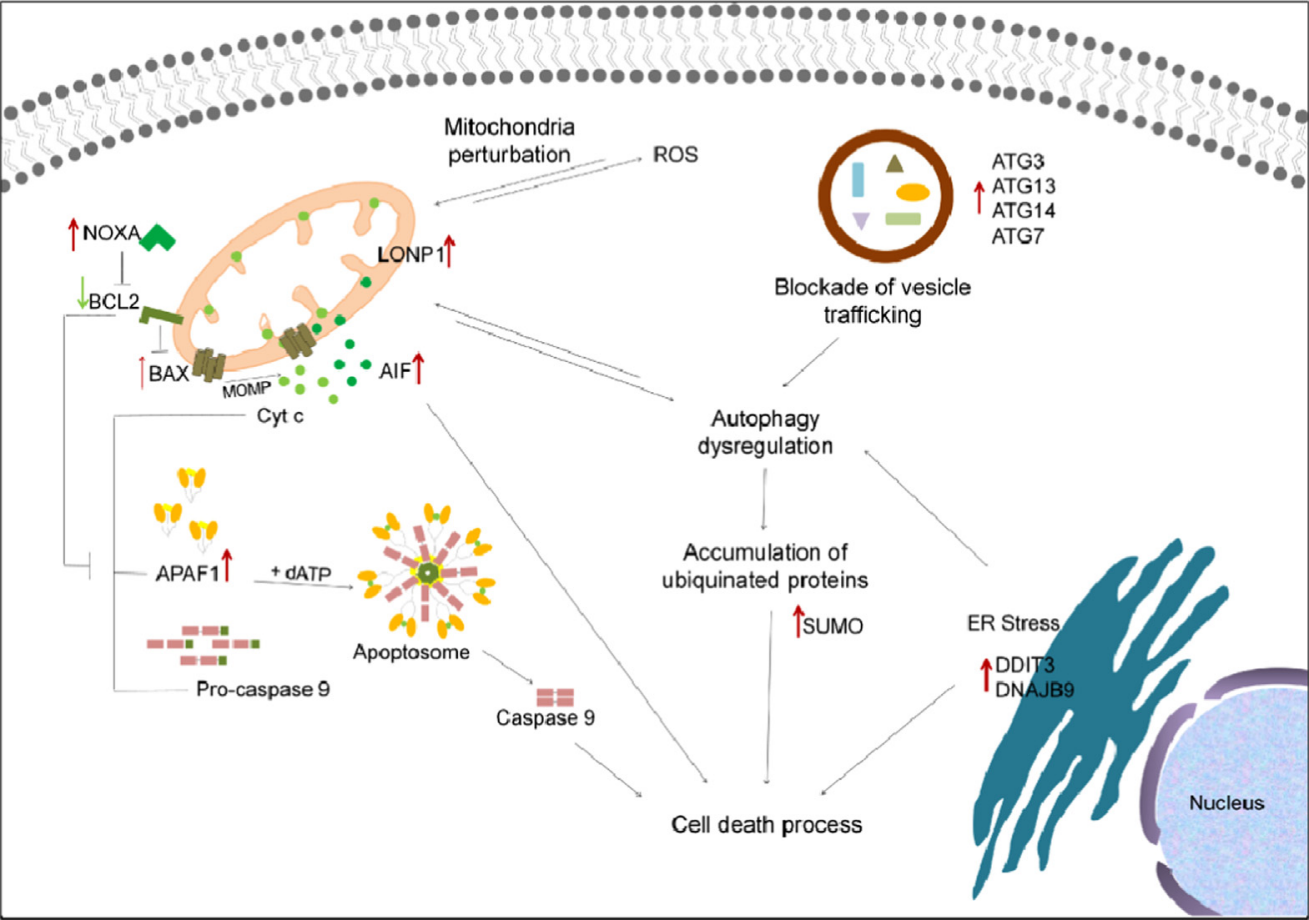
Scheme of the interaction between the intrinsic apoptosis, autophagy and stress response pathways governing the HepG2 response to CdS QDs. Up-regulation is indicated by red arrows and down-regulation by green arrows. Mitochondrial dysfunction is linked to oxidative stress, as the mitochondria are both generators of, and targets for ROS. CdS QDs may undergo autophagic sequestration and then selective compartmentalization in autophagosome, leading to a dysfunctional autophagic pathway due to the inhibition of autophagic flux. The effect could also be associated with an accumulation of ubiquitinated proteins.

The data reported in Table 4 were analysed to build a heat map (Fig. 7) depicting the hierarchical clustering of genes according to their expression profile. Moreover, data were summarized in a scheme that highlights the interactions between the different cellular processes involved (Fig. 8).

## Figures and Tables

**Table 1:** Literature survey detailing parameters of cell functionality determined in experiments with cadmium-based quantum dots.

**Reference**	**Quantum dots**	**Dimensions**	**Cell line(s)**	**Experimental evaluation of cell parameters**	**Main effects or toxicity mechanisms**
Al-Ali et al. [2]	Commercial, CdSe/ZnS coated with hexadecylamine, carboxyl groups or polyethylene glycol	From 4 nm to 82 nm, peak at 6 nm	THP-1 (human monocytic cell line) differentiated into macrophages	Cell viability (Relative population doubling)	No significant cytotoxicity at 24 h, some cytotoxicity at 72 h of exposure. Induction of inflammatory response. Modification of gene expression for genes of oxidative stress pathway
				Inflammatory response (ELISA analysis for cytokine, chemokine)	
				Oxidative stress (Gene expression analysis)	
Alomar [3]	CdS	From 53.40 nm to 5.60 nm	A549 (human lung adenocarcinoma epithelial cells)	Cell viability (MTT assay and neutral red uptake)	Loss of cell viability. Induction of oxidative stress. Reduced mitochondrial function. Induced lysosome activity. Induction of apoptosis and inflammatory response
				Mitochondrial functionality (Mitochondrial membrane potential)	
				Oxidative stress (Intracellular reactive oxygen species (ROS) levels, lipid peroxidation assay, glutathione (GSH) levels, superoxide dismutase activity)	
				Apoptosis (Staining of chromosomes, caspase assay, COMET assay)	
				Inflammatory response (ELISA assay for release of cytokines)	
Bao et al. [4]	CdSe, or CdSe core with CdS shell	6 nm	Five human cell lines: HL-7702, Bel-7402, Bel-7404, 786-O, HeLa	Cell viability (MTT assay)	Cytotoxicity increased by CdS shell, differing among cell lines. Induction of apoptosis. Toxic to mice
				Apoptosis (Flow cytometry)	
				Acute toxicity (mice)	
Hossain and Mukherjee [5]	CdS	3 nm	HeLa (human cell line)	Cell viability (MTT assay, microscopy)	Decrease of cell viability. Accumulation of ROS. Changes in cell and nucleus morphology
				Oxidative stress (intracellular ROS levels)	
Ju et al. [6]	CdSe core with ZnS shell, uncoated or coated with polyethylene glycol	440 nm and 680 nm	HSF-42 (human skin fibroblasts)	Cell viability (MTT assay and Trypan blue exclusion)	Uncoated QDs decrease cell viability, increase foci formation and DNA damage, but without apoptosis. They induce increased ROS levels. Coated QDs do not produce effects
				DNA damage (formation of foci, Comet assay)	
				Apoptosis (Western blot for caspase)	
				Oxidative stress (intracellular ROS levels)	
Li et al. [7]	CdTe with thioglycolic acid	Around 2 nm	NIH/3T3 (mouse cell line)	Cell viability (WST-1 assay)	Release of Cd ions. Decrease in cell viability. Changes in miRNA expression
				miRNA expression (extraction, sequencing, real time PCR analysis, Western blot for p53)	
Luo et al. 2013 [8]	Commercial: Cd/Se core, shell with ZnS	From 8 nm to 12 nm (10±2 nm)	RAG (mouse renal adenocarcinoma)	Cell viability (MTT assay, Lactate Dehydrogenase activity assay to detect necrosis)	Increase in intracellular levels of reactive oxygen species. Induction of autophagy, followed by apoptosis. QDs localised in ER, endosomes, lysosomes, mitochondria. Effect on mitochondrial function. Autophagy induced by oxidative stress is seen as a defense mechanism
				Mitochondrial functionality (ATP level and membrane potential)	
				Apoptosis (TUNEL staining)	
				Autophagy (microscopy and protein analysis)	
Manshian et al. [9]	Commercial CdSe core with ZnS shell, amine or carboxyl functional groups on the surface	From 3 nm to5 nm	Three human cell lines: BEAS-2B, HFF-1, TK6	Cell viability (flow cytometry and microscopy, Relative population doubling)	Cytotoxicity depending on cell types, BEAS-2B showed no effects. Carboxyl functionalised QDs exert genotoxic effects. Minimal increase of ROS levels. Decrease in mitochondrial functionality with carboxyl QDs. No inflammatory effect
				DNA damage (Micronuclei frequency, pancentromeric staining)	
				Oxidative stress (intracellular ROS levels)	
				Mitochondrial functionality (membrane potential)	
				Inflammatory response (ELISA assay for cytokines)	
Nguyen et al. [10]	Commercial, CdTe core, CdS shell, coated by polyacrylate	7.3±1.2 nm	HepG2 (human hepatocellular carcinoma)	Cell viability (MTT assay)	Decrease in cell viability. Swelling of mitochondria and loss of cristae. Localisation of QDs in mitochondria. Loss of membrane potential. Increase in cytosolic calcium levels. Inhibition of respiration. Decrease in ATP levels. Decrease in complexes II, III, IV, no change in complexes I and V. Activation of mitochondrial biogenesis
				Mitochondrial morphology (Transmission Electron Microscopy, TEM)	
				Mitochondrial functionality (membrane potential, quantification of complexes, activity assay, oxygen consumption, ATP levels)	
				Mitochondria biogenesis (ELISA for PGC-1alpha levels)	
				Localisation of QDs (mitochondria enrichment)	
				Oxidative stress and apoptosis (release of calcium)	
Nguyen et al. [11]	Commercial, CdTe core with CdS shell coated with polymers	7.3±1.2 nm	J774A.1 (mouse macrophage), HT29 (human colon epithelium)	Cell viability (MTT assay, microscopy)	Decrease in cell viability. Morphological changes in cells, actin filaments, nucleus. No changes in inflammatory markers. Increase in the cytotoxicity of bacterial infection and alteration of immune response
				Nitric oxide production (Griess reagent)	
				Inflammatory response (cytokines and chemokines assay)	
Nguyen et al. [12]	Commercial, CdTe core with CdS shell coated with polymers	7.3±1.2 nm	HepG2	Cell viability (MTT assay)	Cause oxidative stress, interfere with antioxidant defenses and activate protein kinases, leading to apoptosis via both extrinsic and intrinsic pathways. Effects of CdTe-QDs were similar or greater compared to those of CdCl_2_ at equivalent concentrations of cadmium
				Oxidative stress (intracellular ROS levels, GSH levels, SOD and CAT activity, Nrf2 activation)	
				Apoptosis (caspase activity, Annexin stain, Bcl2 and Bax levels)	
Peng et al. [13]	Four types of commercial QDs, CdSe core and ZnS shell, functional groups on the surface	From 15 nm to 25 nm	HepG2 (human hepatocellular carcinoma)	Cell viability (MTT assay)	Localisation to lysosomes. No decrease in cell viability. No apoptosis. No changes in GSH levels. Upregulation of stress response genes
				Apoptosis (DNA staining)	
				Oxidative stress (GSH levels)	
				Stress response (analysis of gene expression)	
Romoser et al. [14]	Commercial, CdSe/ZnS-COOH	15 nm	HEK (human epidermal keratinocytes), HDF (human dermal fibroblasts)	Cell viability (resazurin assay, microscopy)	Morphological changes and loss of viability. Internalisation of QDs. Upregulation of genes involved in NFkappaB pathway. Induction of inflammatory and immune response. Induction of apoptotic genes. Induction of markers for oxidative stress
				Localisation of QDs (fluorescence)	
				Inflammatory response, immune response, apoptosis (gene expression analysis, Western blot, transcriptional activity of NFkappaB)	
Smith et al. [15]	CdSe core, ZnS shell, coated with polymers TOPO and PMAT	12.7±0.5 nm	HepG2 (human hepatocellular carcinoma)	Localisation of QDs (fluorescence microscopy)	No effect on cell viability. No induction of GSH. No effect on oxidative stress gene markers. No induction of inflammatory response
				Cell viability (MTT assay)	
				Oxidative stress (GSH levels)	
				Stress response (analysis of gene expression, Western blotting)	
				Inflammatory response (cytokine assay)	
Tan et al. [16]	CdSe core, ZnS shell, polymer coating with carboxyl or amine groups, functionalisation	From 20 nm to 30 nm	HepG2 (human hepatocellular carcinoma), NIH/3T3 (mouse cell line)	Cell viability (microscopy, MTT assay)	Effects depend on surface charge, hydrophobicity and presence of PEG. Cytotoxicity depends on intake. QDs with PEG can reach the lysosomes. Endocytosis is mediated by clathrin
				Localisation of QDs (microscopy)	
				Endocytosis (inhibitors)	
Wu et al. [17]	CdTe coated with carboxyl groups	Unspecified	HepG2 (human hepatocellular carcinoma)	Apoptosis (DNA staining)	Induction of apoptosis. Increase of ROS and decrease of GSH. Induction of autophagic vacuoles. Presence of swollen mitochondria
				Oxidative stress (intracellular ROS levels, GSH levels)	
				Autophagy (TEM)	
Xu et al. [18]	CdTe, and nanocomposites of QDs with gambogic acid, GA-CdTe	About 5 nm	HELF (human embryonic lung fibroblasts), HepG2 (human hepatocellular carcinoma)	Cell viability (MTT assay)	Little effect on cell viability for CdTe QDs, enhanced by GA. Intake into cells. Induction of apoptosis not assessed, no changes in cell cycle phases
				Cell morphology (DAPI staining)	
				QDs localisation (fluorescence microscopy)	
				Apoptosis (annexin,propidium iodide, flow cytometry)	
Zhang et al. [19]	CdTe	2.2 nm	AML 12 (mouse hepatocytes)	Cell viability (MTT assay)	Loss of cell viability. Decrease in ATP levels. Induction of oxidative stress. Induction of apoptosis and related genes for mitochondria-dependent pathways. Activation of Nrf2 pathway for response to ROS
				Mitochondrial functionality (ATP levels)	
				Oxidative stress (intracellular ROS levels)	
				Apoptosis (annexin, propidium iodide, flow cytometry, analysis of gene expression, Western blot)	
Zhang et al. [20]	Commercial, CdSe core, ZnS shell, coated with PEG: neutral, acid or basic	core/shell: 6 nm (minor axis) ×12 nm (major axis)	Dendritic cells derived from PBMC (pig peripheral blood mononuclear cells)	Cell viability (flow cytometry, proliferation, cell counting, Alamar-Blue assay)	Dencritic cells intake QDs with acidic surface. Endocytosis involves clathrin and partially micropinocytosis Small loss in cell viability. Induction of oxidative stress. No induction of inflammatory response
				Endocytosis (inhibitors)	
				Oxidative stress (GSH levels)	
				Inflammatory response (cytokine assay)	
Zhang et al. [21]	CdTe coated with 3-mercaptopropionic acid	About 4 nm	HepG2/ADM (human hepatoma, adriamycin resistant cells)	Cell viability (MTT assay, microscopy)	Decrease in cell viability, synergistic effect with drug daunorubicin through facilitated uptake. Induction of apoptosis, DNA fragmentation. Activation of caspases after release of cytochrome C. Inhibition of tumor growth in synergy with drug
				Drug uptake (electrochemical assay)	
				Apoptosis (DNA staining, flow cytometry, DNA fragmentation, Western blots, TUNEL staining)	
				Acute toxicity (mice)	

**Table 2:** A comparisons of the effects of exposure to CdS QDs and Cd^2+^ ions.

	**Gene expression**	**Oxidative Stress**	**Genotoxicity**	**Cell death**
**CdS QDs** (our experiments)	Most of the induced genes belonged to one of the three major functional categories “apoptosis", "autophagy" and "stress response"	CdS QDs did not induce a rapid ROS generation, causing only mild increase in reactive oxygen species (ROS) levels	Exposure to CdS QDs generated, in HepG2 cells, a minor degree of DNA damage; after a major exposure, the extent of the damage was almost indistinguishable from that shown by the non-treated control	The data suggest that mitochondria-mediated intrinsic apoptosis pathway were activated by CdS QDs exposure, but did not arrive at the extreme point (mtDNA distruption, cell death)
**Cadmium**	Cadmium induces at least two types of genes: (1) genes coding for detoxifying and other cytoprotective proteins, i.e. metallothioneins, enzymes of glutathione synthesis, heat shock proteins, zinc transporter proteins; and (2) early genes response proto-oncogenes related to cell proliferation control (*c-FOS*, *c-JUN*, c*-MYC*, *EGR-1*) [22]	Cadmium induces a rapid and transient ROS generation [23], causing formation of superoxide ion and hydrogen peroxide [24]. The major toxic effects of increasing doses of Cd concentration involve decreased antioxidant enzyme levels (superoxide dismutase and glutathione peroxidase) [25]	Cadmium causes DNA fragmentation [23]	Oh and Lim [23] demonstrated that Cd-induced cell death was caspase-dependent
			Several authors report the view that genotoxicity induced by Cd is not a direct effect of the metal, but rather due to the generation of reactive oxygen free radicals and the resulting oxidative stress [26]	
			Cd exhibits remarkable potential to inhibit DNA damage repair, and this has been identified as a major mechanism underlying the carcinogenic potential of Cd [27]	

**Table 3:** Rationale of doses and time for CdS QDs treatment.

**Experiment type**	**Treatments**			**Controls**			
	*Substance*	*Doses*^a^	*Times*^b^	*Untreated*	*Medium+reagent assay*	*Medium+QDs+reagent assay*	*Positive*
Flow cytometry^d^	CdS QDs	100 µg mL^−1^	0–30–60–240 min	X			
Cytotoxicity assay^e^	CdS QDs	0.5–100 µg mL^−1^	24 h	X	X	X	Aflatoxin B
	CdSO_4_	Cd concentration equivalent to QDs					
Gene expression analysis^f^	CdS QDs	3–7–14 µg mL^−1^	24 h	X			
Genotoxicity assay^g^	CdS QDs	1.5 µg mL^−1^	1–4–24 h	X			
Reactive oxygen species with DCFH-DA^h^	CdS QDs	3–7–14 µg mL^−1^	1–4 h	X	X	X	H_2_O_2_
Reactive oxygen species with microscope^i^	CdS QDs	14 µg mL^−1^	4 h	X			H_2_O_2_
Nitric oxide assay^j^	CdS QDs	3–7–14 µg mL^−1^	1–4 h	X	X	X	LPS^c^
Glutathione with DTNB^k^	CdS QDs	3–7–14 µg mL^−1^	24 h	X	X	X	
Mitochondrial function^l^	CdS QDs	14 µg mL^−1^	4 h	X			
mtDNA^m^	CdS QDs	3–7–14 µg mL^−1^	24 h	X			

a The concentrations were chosen on the basis of cell viability assay. Three doses were chosen: a toxic (acute) dose, corresponding to half-maximal inhibitory concentration (IC_50_), a sub-toxic (subacute or upperchronic) dose (IC_30_) and an intermediate (chronic) dose (IC_40_).

b The choice of treatment times includes sampling at 1 and 4 h to highlight the early cell response to CdS QDs, and 24 h to observe the late response or the recovery.

c LPS=Lipopolysaccharide.

d Given the small size of the QDs, the cells were treated with 100 µg mL^−1^ to better observe the possible variation of the parameter SS. In addition, the time-dependent uptake was monitored.

e A dose-response curve was obtained by exposing the cells to doses of QDs for 24 h. This curve was used for IC_50_ determination.

f Three doses were chosen: a toxic, corresponding to IC_50_, a sub-toxic (IC_30_) and an intermediate dose (IC_40_).

g Since, for Comet assay, it is necessary to use a dose that does not cause cytotoxic effects, the cells were exposed to 1.5 µg mL^−1^ CdS QDs. The treatment times were 1 and 4 h, to highlight the early DNA damage attributed to oxidative stress, and 24 h to verify the recovery of the damage.

h DCFH-DA is 2′,7′-dichlorodihydrofluorescein diacetate. Doses used are the same as in point f. Treatment times were reduced as 1 and 4 h are enough to highlight the presence of reactive oxygen species (ROS).

i Because 4 h exposure to 14 µg mL^−1^ CdS QDs induced a large increase in ROS level, this condition was used to monitor ROS formation using fluorescence microscopy. H_2_O_2_ treatment, in conditions that give a good signal, was used as positive control.

j See ROS with DCFH-DA.

k DTNB is 5,5′-dithiobis(2-nitrobenzoic acid). The same conditions used for gene expression analysis were used.

l Because 4 h exposure to 14 µg mL^−1^ CdS QDs induced a large increase in ROS level, this condition was used to analyze mitochondrial integrity.

m The same conditions used for gene expression analysis were used to appreciate the possible change in the mtDNA content, following the alteration of mitochondrial function.

**Table 4:** Gene subjected to transcription profiling using TaqMan^®^ Custom Array Plates. Values in bold face are statistically significant at *p*<0.5. Official full name and function derive from UniProt Knowledgebase (UniProtKB) (accessed in July 2016, www.uniprot.org).

**Categories**	**Symbol**	**Official full name**	**Function**	**Fold change (RQ)**	
				**3 µg mL**^**−1**^	**14 µg mL**^**−1**^
**Apoptosis**	*ABL1*	Tyrosine-protein kinase ABL1	Non-receptor tyrosine-protein kinase that plays a role in many key processes linked to cell growth and survival such as cytoskeleton remodeling in response to extracellular stimuli, cell motility and adhesion, receptor endocytosis, autophagy, DNA damage response and apoptosis. Coordinates actin remodeling through tyrosine phosphorylation of proteins controlling cytoskeleton dynamics	0.565	1.475
	*BAK1*	Bcl-2 homologous antagonist/killer	In the presence of an appropriate stimulus, accelerates programmed cell death by binding to, and antagonizing the anti-apoptotic action of BCL2	1.131	1,632
	*BAX*	Apoptosis regulator BAX	Promotes activation of CASP3, and thereby apoptosis	1.860	**2.397**
	*BBC3 (PUMA)*	Bcl-2-binding component 3	Essential mediator of p53/TP53-dependent and p53/TP53-independent apoptosis	0.806	**3.973**
	*CASP2*	Caspase-2	Involved in the activation cascade of caspases responsible for apoptosis execution. Might function by either activating some proteins required for cell death or inactivating proteins necessary for cell survival	1.127	0.961
	*CASP3*	Caspase-3	Involved in the activation cascade of caspases responsible for apoptosis execution. Cleaves and activates caspase −6, −7 and −9	1.455	0.923
	*CHEK2 (RAD53)*	Serine/threonine-protein kinase Chk2	It is required for checkpoint-mediated cell cycle arrest, activation of DNA repair and apoptosis in response to the presence of DNA double-strand breaks. May also negatively regulate cell cycle progression during unperturbed cell cycles	1.685	1.478
	*DEDD2*	DNA-binding death effector domain-containing protein 2	May play a critical role in death receptor-induced apoptosis and may target CASP8 and CASP10 to the nucleus. May regulate degradation of intermediate filaments during apoptosis. May play a role in the general transcription machinery in the nucleus and might be an important regulator of the activity of GTF3C3	0.716	1.497
	*DIABLO (SMAC)*	Diablo homolog, mitochondrial	Promotes apoptosis by activating caspases in the cytochrome c/Apaf-1/caspase-9 pathway. Acts by opposing the inhibitory activity of inhibitor of apoptosis proteins (IAP). Inhibits the activity of BIRC6/bruce by inhibiting its binding to caspases	1.524	1.679
	*HRK (BID3)*	Activator of apoptosis harakiri	Promotes apoptosis	–	–
	*PIDD1 (LRDD)*	p53-induced death domain-containing protein 1	Promotes apoptosis downstream of the tumor suppressor as component of the DNA damage/stress response pathway that connects p53/TP53 to apoptosis	**0.398**	0.569
	*PMAIP1 (NOXA)*	Phorbol-12-myristate-13-acetate-induced protein 1	Promotes activation of caspases and apoptosis. Promotes mitochondrial membrane changes and efflux of apoptogenic proteins from the mitochondria	**2.340**	**3.874**
	*PYCARD*	Apoptosis-associated speck-like protein containing a CARD	Functions as key mediator in apoptosis and inflammation	**2.648**	**3.425**
	*RAD21*	Double-strand-break repair protein rad21 homolog	Cleavable component of the cohesin complex, involved in chromosome cohesion during cell cycle, in DNA repair, and in apoptosis	1.785	**12.435**
	*XIAP (BIRC4)*	E3 ubiquitin-protein ligase XIAP	Multi-functional protein which regulates not only caspases and apoptosis, but also modulates inflammatory signaling and immunity, copper homeostasis, mitogenic kinase signaling, cell proliferation, as well as cell invasion and metastasis. Acts as a direct caspase inhibitor	0.646	0.684
**DNA repair**	*FANCA*	Fanconi anemia group A protein	DNA repair protein that may operate in a postreplication repair or a cell cycle checkpoint function. May be involved in interstrand DNA cross-link repair and in the maintenance of normal chromosome stability	1.456	1.783
	*MRE11A*	MRE11 homolog A, double strand break repair nuclease	Component of the MRN complex, which plays a central role in double-strand break (DSB) repair, DNA recombination, maintenance of telomere integrity and meiosis	–	–
	*MSH2*	DNA mismatch repair protein Msh2	Component of the post-replicative DNA mismatch repair system (MMR). Forms two different heterodimers which bind to DNA mismatches thereby initiating DNA repair	**3.320**	**2.310**
	*NBN*	Nibrin	Component of the MRN complex which plays a critical role in the cellular response to DNA damage and the maintenance of chromosome integrity. The complex is involved in double-strand break repair, DNA recombination, maintenance of telomere integrity, cell cycle checkpoint control and meiosis. The complex possesses single-strand endonuclease activity and double-strand-specific 3׳-5׳ exonuclease activity, which are provided by MRE11A	**3.628**	1.962
	*PRKDC*	DNA-dependent protein kinase catalytic subunit	Serine/threonine-protein kinase that acts as a molecular sensor for DNA damage. Involved in DNA nonhomologous end joining (NHEJ) required for double-strand break repair	1.264	1.239
	*RAD18*	E3 ubiquitin-protein ligase RAD18	E3 ubiquitin-protein ligase involved in postreplication repair of UV-damaged DNA. Postreplication repair functions in gap-filling of a daughter strand on replication of damaged DNA. Associates to the E2 ubiquitin conjugating enzyme UBE2B to form the UBE2B-RAD18 ubiquitin ligase complex involved in mono-ubiquitination of DNA-associated PCNA on ׳Lys-164׳. Has ssDNA binding activity	1.912	1.090
	*REV1*	DNA repair protein REV1	Deoxycytidyl transferase involved in DNA repair. Transfers a dCMP residue from dCTP to the 3׳-end of a DNA primer in a template-dependent reaction	1.615	**2.345**
	*RPA1*	Replication protein A 70 kDa DNA-binding subunit	As part of the heterotrimeric replication protein A complex (RPA/RP-A), binds and stabilizes single-stranded DNA intermediates, which form during DNA replication or upon DNA stress. It prevents their reannealing and in parallel, recruits and activates different proteins and complexes involved in DNA metabolism. Thereby, it plays an essential role both in DNA replication and the cellular response to DNA damage	1.610	1.377
	*XPA*	DNA repair protein complementing XP-A cells	Involved in DNA excision repair. Initiates repair by binding to damaged sites with various affinities, depending on the photoproduct and the transcriptional state of the region	**2.305**	1.409
	*XRCC6*	X-ray repair cross-complementing protein 6e	Single-stranded DNA-dependent ATP-dependent helicase. Has a role in chromosome translocation	1.659	1.958
**Cell cycle**	*BUB3*	Mitotic checkpoint protein BUB3	Has a dual function in spindle-assembly checkpoint signaling and in promoting the establishment of correct kinetochore-microtubule (K-MT) attachments. Promotes the formation of stable end-on bipolar attachments. Necessary for kinetochore localization of BUB1	**2.882**	**3.705**
	*ICMT*	Protein-S-isoprenylcysteine O-methyltransferase	Catalyzes the post-translational methylation of isoprenylated C-terminal cysteine residues	0.983	0.786
	*MDM2*	E3 ubiquitin-protein ligase Mdm2	E3 ubiquitin-protein ligase that mediates ubiquitination of p53/TP53, leading to its degradation by the proteasome. Inhibits p53/TP53- and p73/TP73-mediated cell cycle arrest and apoptosis by binding its transcriptional activation domain	1.177	0.802
	*UBAC1*	Ubiquitin-associated domain-containing protein 1	Non-catalytic subunit of the KPC complex that acts as E3 ubiquitin-protein ligase. Required for poly-ubiquitination and proteasome-mediated degradation of CDKN1B during G1 phase of the cell cycle	1.840	**2.490**
	*ATP5L*	ATP synthase subunit g, mitochondrial	Mitochondrial membrane ATP synthase	**6.887**	**3.234**
	*COX8A*	Cytochrome C oxidase subunit 8A, mitochondrial	This protein is one of the nuclear-coded polypeptide chains of cytochrome C oxidase, the terminal oxidase in mitochondrial electron transport	**2.053**	**2.143**
	*FIS1*	Mitochondrial fission 1 protein	Involved in the fragmentation of the mitochondrial network and its perinuclear clustering. Plays a minor role in the recruitment and association of the fission mediator dynamin-related protein 1 (DNM1L) to the mitochondrial surface and mitochondrial fission. Can induce cytochrome c release from the mitochondrion to the cytosol, ultimately leading to apoptosis	1.158	**2.059**
**Mitochondrial processes**	*MSRB2*	Methionine sulfoxide reductase B2, mitochondrial	Methionine-sulfoxide reductase that specifically reduces methionine (R)-sulfoxide back to methionine. Upon oxidative stress, may play a role in the preservation of mitochondrial integrity by decreasing the intracellular reactive oxygen species build-up through its scavenging role, hence contributing to cell survival and protein maintenance	**2.371**	**3.209**
	*SDHB*	Succinate dehydrogenase [ubiquinone] iron-sulfur subunit, mitochondrial	Iron-sulfur protein (IP) subunit of succinate dehydrogenase that is involved in complex II of the mitochondrial electron transport chain and is responsible for transferring electrons from succinate to ubiquinone (coenzyme Q)	**3.340**	**3.911**
	*PRELID3A (SLMO1)*	PRELI domain containing protein 3A	in vitro, the TRIAP1:PRELID3A complex mediates the transfer of phosphatidic acid (PA) between liposomes and probably functions as a PA transporter across the mitochondrion intermembrane space. Phosphatidic acid import is required for cardiolipin (CL) synthesis in the mitochondrial inner membrane	0.917	0.707
	*UQCR11*	Cytochrome b-c1 complex subunit 10	This is a component of the ubiquinol-cytochrome c reductase complex (complex III or cytochrome b-c1 complex), which is part of the mitochondrial respiratory chain. This protein may be closely linked to the iron-sulfur protein in the complex and function as an iron-sulfur protein binding factor	1.201	**2.129**
	*UQCRC1*	Cytochrome b-c1 complex subunit 1, mitochondrial	This is a component of the ubiquinol-cytochrome c reductase complex (complex III or cytochrome b-c1 complex), which is part of the mitochondrial respiratory chain. This protein may mediate formation of the complex between cytochromes c and c1	1.177	1.859
	*UQCRC2*	Cytochrome b-c1 complex subunit 2, mitochondrial	This is a component of the ubiquinol-cytochrome c reductase complex (complex III or cytochrome b-c1 complex), which is part of the mitochondrial respiratory chain. The core protein 2 is required for the assembly of the complex	**5.311**	**4.399**
	*SUMO1*	Small ubiquitin-related modifier 1	Ubiquitin-like protein that can be covalently attached to proteins as a monomer or a lysine-linked polymer. This plays a crucial role in a number of cellular processes such as nuclear transport, DNA replication and repair, mitosis and signal transduction	**2.429**	**4.445**
**Metabolism of protein**	*UBQLN1*	Ubiquilin 1	Plays an important role in the regulation of different protein degradation mechanisms and pathways including ubiquitin-proteasome system (UPS), autophagy and endoplasmic reticulum-associated protein degradation (ERAD) pathway. Mediates the proteasomal targeting of misfolded or accumulated proteins for degradation by binding (via UBA domain) to their polyubiquitin chains and by interacting (via ubiquitin-like domain) with the subunits of the proteasome. Involved in the regulation of macroautophagy and autophagosome formation; required for maturation of autophagy-related protein LC3 from the cytosolic form LC3-I to the membrane-bound form LC3-II and may assist in the maturation of autophagosomes to autolysosomes by mediating autophagosome-lysosome fusion	1.698	**2.148**
**Metabolism**	*ADH1*	Alcohol dehydrogenase 1A	Catalyzes: an alcohol + NAD+= an aldehyde or ketone + NADH	**3.122**	0.775
	*ACSL4*	Long-chain-fatty-acid–CoA ligase 4	Activation of long-chain fatty acids for both synthesis of cellular lipids, and degradation via beta-oxidation. Preferentially uses arachidonate and eicosapentaenoate as substrates.	1.137	**2.912**
	*DHRS11*^a^	Dehydrogenase/reductase SDR family member 11	It is a member of the large short-chain dehydrogenase/reductase (SDR) family of enzymes that metabolize steroid hormones, prostaglandins, retinoids, lipids, and xenobiotics	0.792	0.889
	*GBP2*	Guanylate-binding protein 2	Catalyzes the hydrolysis GTP to GMP in two consecutive cleavage reactions	**5.811**	**17.782**
	*GCLC*	Glutamate-cysteine ligase catalytic subunit	Involved in glutathione biosynthesis. Catalyzes: ATP+L-glutamate+L-cysteine=ADP+phosphate+gamma-L-glutamyl-L-cysteine	1.194	**3.335**
	*MDH1*	Malate dehydrogenase, cytoplasmic	Catalyzes: (S)-malate +NAD+=oxaloacetate +NADH	**3.679**	**2.884**
	*MPV17*	Protein Mpv17	Involved in mitochondria homeostasis. May be involved in the metabolism of reactive oxygen species and control of oxidative phosphorylation and mitochondrial DNA maintenance	1.484	1.862
	*NAA25*	N(alpha)-acetyltransferase 25, NatB auxiliary subunit	Non-catalytic subunit of the NatB complex which catalyzes acetylation of the N-terminal methionine residues of peptides beginning with Met-Asp-Glu. May play a role in normal cell-cycle progression	**2.677**	**2.176**
	*PDHB*	Pyruvate dehydrogenase E1 component subunit beta, mitochondrial	Catalyzes the overall conversion of pyruvate to acetyl-CoA and CO_2_, and thereby links the glycolytic pathway to the tricarboxylic cycle	1.711	**2.347**
	*PFKP*	ATP-dependent 6-phosphofructokinase, platelet type	Catalyzes the phosphorylation of D-fructose 6-phosphate to fructose 1,6-bisphosphate by ATP, the first committing step of glycolysis	1.672	**3.912**
	*RRM1*	Ribonucleoside-diphosphate reductase large subunit	Provides the precursors necessary for DNA synthesis. Catalyzes the biosynthesis of deoxyribonucleotides from the corresponding ribonucleotides	1.828	1.149
**Stress response**	*DDIT3*	DNA damage-inducible transcript 3 protein	Multifunctional transcription factor in ER stress response. Plays an essential role in the response to a wide variety of cell stresses and induces cell cycle arrest and apoptosis in response to ER stress	**0.485**	**5.474**
	*DNAJB9 (HSP40)*	DnaJ homolog subfamily B member 9	Involved in endoplasmic reticulum-associated degradation (ERAD) of misfolded proteins. Acts as a co-chaperone with an Hsp70 protein	**2.354**	1.929
	*GPX3*	Glutathione peroxidase 3	Protects cells and enzymes from oxidative damage, by catalyzing the reduction of hydrogen peroxide, lipid peroxides and organic hydroperoxide, by glutathione	0.769	**2.162**
	*HSP90AA1*	Heat shock protein HSP 90-alpha	Molecular chaperone that promotes the maturation, structural maintenance and proper regulation of specific target proteins involved for instance in cell cycle control and signal transduction. Undergoes a functional cycle that is linked to its ATPase activity. This cycle probably induces conformational changes in the client proteins, thereby causing their activation. Interacts dynamically with various co-chaperones that modulate its substrate recognition, ATPase cycle and chaperone function	**4.360**	1.074
	*HSPB3*	Heat shock protein beta-3	Inhibitor of actin polymerization	0.830	1.177
	*HSPD1*	60 kDa heat shock protein, mitochondrial	Implicated in mitochondrial protein import and macromolecular assembly. May facilitate the correct folding of imported proteins. May also prevent misfolding and promote the refolding and proper assembly of unfolded polypeptides generated under stress conditions in the mitochondrial matrix	**2.378**	**2.505**
	*HSPH1*	Heat shock protein 105 kDa	Prevents the aggregation of denatured proteins in cells under severe stress, on which the ATP levels decrease markedly. Inhibits HSPA8/HSC70 ATPase and chaperone activities	1.291	0.801
	*LONP1*	Lon protease homolog, mitochondrial	ATP-dependent serine protease that mediates the selective degradation of misfolded, unassembled or oxidatively damaged polypeptides as well as certain short-lived regulatory proteins in the mitochondrial matrix. May also have a chaperone function in the assembly of inner membrane protein complexes. Participates in the regulation of mitochondrial gene expression and in the maintenance of the integrity of the mitochondrial genome. Binds to mitochondrial promoters and RNA in a single-stranded, site-specific, and strand-specific manner. May regulate mitochondrial DNA replication and/or gene expression using site-specific, single-stranded DNA binding to target the degradation of regulatory proteins binding to adjacent sites in mitochondrial promoters	**3.157**	**3.041**
	*SIRT1*	NAD-dependent protein deacetylase sirtuin-1	NAD-dependent protein deacetylase that links transcriptional regulation directly to intracellular energetics and participates in the coordination of several separated cellular functions such as cell cycle, response to DNA damage, metobolism, apoptosis and autophagy	1.008	1.838
	*TXN2*	Thioredoxin, mitochondrial	Has an anti-apoptotic function and plays an important role in the regulation of mitochondrial membrane potential. Could be involved in the resistance to anti-tumor agents. Possesses a dithiol-reducing activity	1.978	1.225
**miRNA biogenesis**	*DROSHA*	Ribonuclease 3	Ribonuclease III double-stranded (ds) RNA-specific endoribonuclease that is involved in the initial step of microRNA (miRNA) biogenesis	**2.512**	1.790
	*DICER1*	Endoribonuclease Dicer	Double-stranded RNA (dsRNA) endoribonuclease playing a central role in short dsRNA-mediated post-transcriptional gene silencing	1.495	0.863
	*AGO2 (EIF2C2)*	Protein argonaute-2	Required for RNA-mediated gene silencing (RNAi) by the RNA-induced silencing complex (RISC)	1.109	**2.170**
	*XPO5*	Exportin-5	Mediates the nuclear export of proteins bearing a double-stranded RNA binding domain (dsRBD) and double-stranded RNAs (cargos)	1.452	1.541
**Gene expression**	*DOM3Z*	Decapping and exoribonuclease protein	Ribonuclease that specifically degrades pre-mRNAs with a defective 5׳ end cap and is part of a pre-mRNA capping quality control	1.734	**2.558**
	*METTL18*	Histidine protein methyltransferase 1 homolog	Probable histidine methyltransferase	**2.395**	**2.295**
	*MRPL44*	39S ribosomal protein L44, mitochondrial	Component of the 39S subunit of mitochondrial ribosome. May have a function in the assembly/stability of nascent mitochondrial polypeptides exiting the ribosome	**2.670**	**2.392**
	*MTIF2*	Translation initiation factor IF-2, mitochondrial	One of the essential components for the initiation of protein synthesis. Protects formylmethionyl-tRNA from spontaneous hydrolysis and promotes its binding to the 30S ribosomal subunits. Also involved in the hydrolysis of GTP during the formation of the 70S ribosomal complex.	**3.941**	**2.292**
	*N6AMT1*	HemK methyltransferase family member 2	Heterodimeric methyltransferase that catalyzes N5-methylation of ETF1 on ׳Gln-185׳, using S-adenosyl L-methionine as methyl donor. ETF1 needs to be complexed to ERF3 in its GTP-bound form to be efficiently methylated	**6.317**	**2.233**
	*RPL36AL*	60S ribosomal protein L36a-like	Structural constituent of ribosome	–	–
	*RPS8*	40S ribosomal protein S8	Structural constituent of ribosome	**2.537**	**4.553**
	*RTF1*	RNA polymerase-associated protein RTF1 homolog	Component of the PAF1 complex which has multiple functions during transcription by RNA polymerase II	**2.562**	**2.184**
	*SUPT3H*	Transcription initiation protein SPT3 homolog	Probable transcriptional activator	**4.612**	**8.239**
	*TADA1*	Transcriptional adapter 1	Probably involved in transcriptional regulation	1.719	1.796
	*XRN2*	5׳-3׳ exoribonuclease 2	May promote the termination of transcription by RNA polymerase II. During transcription termination, cleavage at the polyadenylation site liberates a 5׳ fragment which is subsequently processed to form the mature mRNA and a 3׳ fragment which remains attached to the elongating polymerase	**2.860**	**3.754**
	*YARS2*	Tyrosyl-tRNA synthetase, mitochondrial	Catalyzes the attachment of tyrosine to tRNA(Tyr) in a two-step reaction: tyrosine is first activated by ATP to form Tyr-AMP and then transferred to the acceptor end of tRNA(Tyr)	**3.901**	**3.562**
	*ZNF180*	Zinc finger protein 180	May be involved in transcriptional regulation	1.010	0.680
**Others**	*ACE*	Angiotensin-converting enzyme	Converts angiotensin I to angiotensin II by release of the terminal His-Leu, this results in an increase of the vasoconstrictor activity of angiotensin. Also able to inactivate bradykinin, a potent vasodilator. Has also a glycosidase activity which releases GPI-anchored proteins from the membrane by cleaving the mannose linkage in the GPI moiet	–	–
	*ATG3*	Ubiquitin-like-conjugating enzyme ATG3	E2 conjugating enzyme required for the cytoplasm to vacuole transport, autophagy, and mitochondrial homeostasis. Responsible for the E2-like covalent binding of phosphatidylethanolamine to the C-terminal Gly of ATG8-like proteins (GABARAP or MAP1LC3A). The formation of the ATG8-phosphatidylethanolamine conjugates is essential for autophagy and for the cytoplasm to vacuole transport. Preferred substrate is MAP1LC3A	**3.323**	**2.078**
	*ATP6V1E1*	V-type proton ATPase subunit E 1	Subunit of the peripheral V1 complex of vacuolar ATPase essential for assembly or catalytic function. V-ATPase is responsible for acidifying a variety of intracellular compartments in eukaryotic cells	**3.972**	**3.311**
	*CHAC2*	Putative glutathione-specific gamma-glutamylcyclotransferase 2	Catalyzes the cleavage glutathione into 5-oxoproline and a Cys-Gly dipeptide. Acts specifically on glutathione, but not on other gamma-glutamyl peptides	**2.163**	0.596
	*ESD*	S-formylglutathione hydrolase	Serine hydrolase involved in the detoxification of formaldehyde	**3.820**	**3.603**
	*FNBP1L*	Formin-binding protein 1-like	Required to coordinate membrane tubulation with reorganization of the actin cytoskeleton during endocytosis. May bind to lipids such as phosphatidylinositol 4,5-bisphosphate and phosphatidylserine and promote membrane invagination and the formation of tubules	1.845	**2.754**
	*GAD1*	Glutamate decarboxylase 1	Catalyzes the production of GABA	**2.746**	**10.255**
	*HEMK1*	HemK methyltransferase family member 1	N5-glutamine methyltransferase responsible for the methylation of the GGQ triplet of the mitochondrial translation release factor MTRF1L	1.119	1.354
	*HSPA8*	Heat shock 71 kDa protein	Acts as a repressor of transcriptional activation. Inhibits the transcriptional coactivator activity of CITED1 on Smad-mediated transcription. Chaperone. Component of the PRP19-CDC5L complex that forms an integral part of the spliceosome and is required for activating pre-mRNA splicing. May have a scaffolding role in the spliceosome assembly as it contacts all other components of the core complex	0.830	1.177
	*LCP1*	Plastin-2	Actin-binding protein	**0.342**	1.324
	*NEDD8*	NEDD8 (Neural precursor cell expressed, developmentally down-regulated 8)	Ubiquitin-like protein which plays an important role in cell cycle control. Covalent attachment to its substrates requires prior activation by the E1 complex UBE1C-APPBP1 and linkage to the E2 enzyme UBE2M. Attachment of NEDD8 to cullins activates their associated E3 ubiquitin ligase activity, and thus promotes polyubiquitination and proteasomal degradation of cyclins and other regulatory proteins	1.866	1.834
	*PEX7*	Peroxisomal targeting signal 2 receptor	Binds to the N-terminal PTS2-type peroxisomal targeting signal and plays an essential role in peroxisomal protein import	**7.444**	**2.405**
	*PHB2*	Prohibitin-2	Acts as a mediator of transcriptional repression by nuclear hormone receptors via recruitment of histone deacetylases	1.730	1.807
	*STX16*	Syntaxin-16	SNARE involved in vesicular transport from the late endosomes to the trans-Golgi network	1.352	**2.038**
	*TWF1*	Twinfilin-1	Actin-binding protein involved in motile and morphological processes. Inhibits actin polymerization	**3.656**	**2.781**
**Candidate endogenous**	*18S*	Eukaryotic 18S rRNA	–		
	*ACTB*	Actin, beta	–		
	*GAPDH*	Glyceraldehyde-3-phosphate dehydrogenase	–		

a The description of gene function has been taken from OMIM® database (accessed in July 2016, www.omim.org).

**Table 5:** Gene subjected to transcription profiling by qRT-PCR (SYBR^®^ Green)*.* Values in bold face are statistically significant at *p*<0.5. Official full name and function derive from UniProt Knowledgebase (UniProtKB) (accessed in July 2016, www.uniprot.org).

**Categories**	**Symbol**	**Official Full Name**	**Function**	**Fold Change (RQ)**		
				**3 µg mL**^**−1**^	**7 µg mL**^**−1**^	**14 µg mL**^**−1**^
**Apoptosis**	*AIFM2*	Apoptosis-inducing factor 2	Oxidoreductase, which may play a role in mediating a p53/TP53-dependent apoptosis response. Probable oxidoreductase that acts as a caspase-independent mitochondrial effector of apoptotic cell death	**5.938**	1.002	**0.497**
	*CASP7*	Caspase 7	Involved in the activation cascade of caspases responsible for apoptosis execution	**0.448**	0.501	**0.349**
	*DAPK1*	Death-associated protein kinase 1	Calcium/calmodulin-dependent serine/threonine kinase involved in multiple cellular signaling pathways that trigger cell survival, apoptosis, and autophagy. Regulates both type I apoptotic and type II autophagic cell deaths signal, depending on the cellular setting. The former is caspase-dependent, while the latter is caspase-independent and is characterized by the accumulation of autophagic vesicles	0.644	0.809	0.709
	*FADD*	FAS-associated death domain protein	Apoptotic adaptor molecule that recruits caspase-8 or caspase-10 to the activated Fas (CD95) or TNFR-1 receptors	**5.776**	0.735	0.660
	*MAP3K5*	Mitogen-activated protein kinase kinase kinase 5	Serine/threonine kinase which acts as an essential component of the MAP kinase signal transduction pathway. Mediates signaling for determination of cell fate such as differentiation and survival. Plays an important role in the cascades of cellular responses evoked by changes in the environment. Plays a crucial role in the apoptosis signal transduction pathway through mitochondria-dependent caspase activation	**0.192**	**0.037**	**0.205**
	*PYCARD*	Apoptosis-associated speck-like protein containing a CARD	Functions as key mediator in apoptosis and inflammation. Promotes caspase-mediated apoptosis involving predominantly caspase-8 and also caspase-9 in a probable cell type-specific manner. Involved in activation of the mitochondrial apoptotic pathway, promotes caspase-8-dependent proteolytic maturation of BID independently of FADD in certain cell types and also mediates mitochondrial translocation of BAX and activates BAX-dependent apoptosis coupled to activation of caspase−9, −2 and −3	1.270	1.510	0.793
	*BAD*	Bcl2-associated agonist of cell death	Promotes cell death. Successfully competes for the binding to Bcl-X(L), Bcl-2 and Bcl-W, thereby affecting the level of heterodimerization of these proteins with BAX	**3.000**	**0.487**	0.559
	*BCL2*	Apoptosis regulator Bcl-2	Regulates cell death by controlling the mitochondrial membrane permeability. Appears to function in a feedback loop system with caspases. Inhibits caspase activity either by preventing the release of cytochrome c from the mitochondria and/or by binding to the apoptosis-activating factor (APAF-1)	0.521	**0.375**	**0.204**
	*APAF1*	Apoptotic protease-activating factor 1	Oligomeric Apaf-1 mediates the cytochrome c-dependent autocatalytic activation of pro-caspase-9 (Apaf-3), leading to the activation of caspase-3 and apoptosis	**5.464**	0.844	**0.423**
	*BAK1*	Bcl-2 homologous antagonist/killer	In the presence of an appropriate stimulus, accelerates programmed cell death by binding to, and antagonizing the anti-apoptotic action of BCL2 or its adenovirus homolog E1B 19k protein	**0.357**	**0.429**	**0.421**
	*BID*	BH- interacting domain death agonist	The major proteolytic product p15 BID allows the release of cytochrome C	0.505	0.797	**0.369**
	*CASP8*	Caspase-8	Most upstream protease of the activation cascade of caspases responsible for the TNFRSF6/FAS mediated and TNFRSF1A induced cell death. Binding to the adapter molecule FADD recruits it to either receptor	**0.473**	0.684	**0.326**
	*CASP9*	Caspase-9	Involved in the activation cascade of caspases responsible for apoptosis execution. Binding of caspase-9 to Apaf-1 leads to activation of the protease which then cleaves and activates caspase-3. Promotes DNA damage-induced apoptosis in a ABL1/c-Abl-dependent manner. Proteolytically cleaves poly(ADP-ribose) polymerase	1.172	**2.585**	1.021
	*AOX1*	Aldehyde oxidase	Plays a key role in the metabolism of xenobiotics and drugs	**10.629**	0.807	1.169
**Stress response**	*COX4I1*	Cytochrome c oxidase subunit 4 isoform 1, mitochondrial	This protein is one of the nuclear-coded polypeptide chains of cytochrome c oxidase, the terminal oxidase in mitochondrial electron transport	1.374	1.034	1.038
	*CAT*	Catalase	Occurs in almost all aerobically respiring organisms and serves to protect cells from the toxic effects of hydrogen peroxide	1.705	0.714	1.873
	*CYGB*	Cytoglobin	May have a protective function during conditions of oxidative stress	**4.563**	0.514	0.509
	*DHCR24*	Delta(24)-sterol reductase	Catalyzes the reduction of the delta-24 double bond of sterol intermediates. Protects cells from oxidative stress by reducing caspase 3 activity during apoptosis induced by oxidative stress	**3.824**	0.545	0.807
	*DUSP1*	Dual specificity phosphatase 1	Induction by oxidative stress and heat shock. Dual specificity phosphatase that dephosphorylates MAP kinase MAPK1/ERK2 on both ׳Thr-183׳ and ׳Tyr-185׳, regulating its activity during the meiotic cell cycle	1.045	0.962	0.966
	*FOXO1*	Forkhead box protein O1	Transcription factor that is the main target of insulin signaling and regulates metabolic homeostasis in response to oxidative stress	1.467	0.825	1.108
	*FOXO3*	Forkhead box protein O3	Transcriptional activator which triggers apoptosis in the absence of survival factors, including neuronal cell death upon oxidative stress	0.986	1.076	1.354
	*GPX1*^a^	Glutathione peroxidase 1	This gene encodes a member of the glutathione peroxidase family. Glutathione peroxidase functions in the detoxification of hydrogen peroxide	1.197	1.592	1.153
	*GPX4*	Phospholipid hydroperoxide glutathione peroxidase, mitochondrial	Protects cells against membrane lipid peroxidation and cell death. Protects from radiation and oxidative damage	0.902	1.269	0.889
	*HIF1A*	Hypoxia inducible factor 1-alpha	Functions as a master transcriptional regulator of the adaptive response to hypoxia. Under hypoxic conditions, activates the transcription of over 40 genes, including erythropoietin, glucose transporters, glycolytic enzymes, vascular endothelial growth factor, HILPDA, and other genes whose protein products increase oxygen delivery or facilitate metabolic adaptation to hypoxia	0.990	1.039	**2.767**
	*MSRA*	Mitochondrial peptide methionine sulfoxide reductase	It has an important function as a repair enzyme for proteins that have been inactivated by oxidation	**4.098**	1.161	**0.369**
	*OXSR1*	Serine/threonine-protein kinase OSR1	Regulates downstream kinases in response to environmental stress. May also have a function in regulating the actin cytoskeleton	**2.780**	0.930	0.593
	*PRDX1*	Peroxiredoxin-1	Involved in redox regulation of the cell. Reduces peroxides with reducing equivalents provided through the thioredoxin system but not from glutaredoxin. May play an important role in eliminating peroxides generated during metabolism	0.835	0.883	0.530
	*PRDX2*	Peroxiredoxin-2	Involved in redox regulation of the cell. Reduces peroxides with reducing equivalents provided through the thioredoxin system. It is not able to receive electrons from glutaredoxin	0.966	1.071	1.035
	*PRDX3*	Thioredoxin-dependent peroxide reductase, mitochondrial	Involved in redox regulation of the cell. Protects radical-sensitive enzymes from oxidative damage by a radical-generating system	**6.320**	**2.204**	0.812
	*PRDX5*	Peroxiredoxin-5, mitochondrial	Reduces hydrogen peroxide and alkyl hydroperoxides with reducing equivalents provided through the thioredoxin system. Involved in intracellular redox signaling	1.424	0.541	0.527
	*PRDX6*	Peroxiredoxin-6	Involved in redox regulation of the cell. Can reduce H_2_O_2_ and short chain organic, fatty acid, and phospholipid hydroperoxides. May play a role in the regulation of phospholipid turnover as well as in protection against oxidative injury	1.860	0.774	0.509
	*PTGS1*	Prostaglandin G/H synthase 1	Converts arachidonate to prostaglandin H2 (PGH2), a committed step in prostanoid synthesis	**0.285**	**0.144**	0.816
	*GBP2*	Guanylate-binding protein 2	Hydrolyzes GTP to GMP in two consecutive cleavage reactions	0.901	0.868	1.959
	*STK25*	Serine/threonine protein kinase 25	Oxidant stress-activated serine/threonine kinase that may play a role in the response to environmental stress. Targets to the Golgi apparatus where it appears to regulate protein transport events, cell adhesion, and polarity complexes important for cell migration	0.561	0.516	0.646
	*GSR*	Glutathione reductase, mitochondrial	Maintains high levels of reduced glutathione in the cytosol	**2.246**	**2.211**	**3.446**
	*GSTZ1*	Maleylacetoacetate isomerase (Glutathione S-transferase zeta 1)	Bifunctional enzyme showing minimal glutathione-conjugating activity with ethacrynic acid and 7-chloro-4-nitrobenz-2-oxa-1,3-diazole and maleylacetoacetate isomerase activity. Has also low glutathione peroxidase activity with T-butyl and cumene hydroperoxides. Is able to catalyze the glutathione dependent oxygenation of dichloroacetic acid to glyoxylic acid	**5.637**	0.644	**3.655**
	*GCLC*	Glutamate-cysteine ligase, catalytic subunit	This protein is involved in step 1 of the subpathway that synthesizes glutathione from L-cysteine and L-glutamate	**0.235**	**0.483**	**0.241**
	*GSS*	Glutathione synthetase	This protein is involved in step 2 of the subpathway that synthesizes glutathione from L-cysteine and L-glutamate	**3.095**	**0.467**	1.625
	*LONP1*	Lon protease homolog, mitochondrial	ATP-dependent serine protease that mediates the selective degradation of misfolded, unassembled or oxidatively damaged polypeptides as well as certain short-lived regulatory proteins in the mitochondrial matrix. May also have a chaperone function in the assembly of inner membrane protein complexes. Participates in the regulation of mitochondrial gene expression and in the maintenance of the integrity of the mitochondrial genome. Binds to mitochondrial promoters and RNA in a single-stranded, site-specific, and strand-specific manner. May regulate mitochondrial DNA replication and/or gene expression using site-specific, single-stranded DNA binding to target the degradation of regulatory proteins binding to adjacent sites in mitochondrial promoters	0.829	1.021	0.635
	*MPV17*	Protein Mpv17	Involved in mitochondria homeostasis. May be involved in the metabolism of reactive oxygen species and control of oxidative phosphorylation and mitochondrial DNA maintenance	1.090	**0.389**	**0.470**
	*NOS2*	Nitric oxide synthase, inducible	Produces nitric oxide (NO) which is a messenger molecule with diverse functions throughout the body.	1.602	**0.334**	**0.408**
	*OXR1*	Oxidation resistance protein 1	May be involved in protection from oxidative damage	**3.543**	0.966	0.635
	*SOD1*	Superoxide dismutase [Cu-Zn]	Destroys radicals which are normally produced within the cells and which are toxic to biological systems	1.747	1.108	0.938
	*SOD2*	Superoxide dismutase [Mn], mitochondrial	Destroys superoxide anion radicals which are normally produced within the cells and which are toxic to biological systems	0.998	0.567	0.995
	*TXN*	Thioredoxin	Participates in various redox reactions through the reversible oxidation of its active center dithiol to a disulfide and catalyzes dithiol-disulfide exchange reactions. Plays a role in the reversible S-nitrosylation of cysteine residues in target proteins, and thereby contributes to the response to intracellular nitric oxide. Nitrosylates the active site Cys of CASP3 in response to nitric oxide (NO), and thereby inhibits caspase-3 activity	0.914	1.089	1.478
	*TXNRD2*	Thioredoxin reductase 2, mitochondrial	Maintains thioredoxin in a reduced state. Implicated in the defenses against oxidative stress. May play a role in redox-regulated cell signaling	1.400	**0.434**	**0.338**
	*UCP2*	Mitochondrial uncoupling protein 2	UCP are mitochondrial transporter proteins that create proton leaks across the inner mitochondrial membrane, thus uncoupling oxidative phosphorylation from ATP synthesis. As a result, energy is dissipated in the form of heat	0.597	0.775	0.715
**Autophagy**	*ATG12*	Ubiquitin-like protein ATG12	Ubiquitin-like protein involved in autophagy vesicles formation. Conjugation with ATG5 through a ubiquitin-like conjugating system involving also ATG7 as an E1-like activating enzyme and ATG10 as an E2-like conjugating enzyme, is essential for its function	1.600	0.581	0.747
	*ATG13*	Autophagy-related protein 13	Autophagy factor required for autophagosome formation and mitophagy. Target of the TOR kinase signaling pathway that regulates autophagy through the control of the phosphorylation status of ATG13 and ULK1	**4.423**	**0.463**	0.588
	*ATG14*	Beclin 1-associated autophagy-related key regulator	Required for both basal and inducible autophagy. Plays a role in autophagosome formation	**6.869**	0.821	0.561
	*ATG7*	Ubiquitin-like modifier-activating enzyme ATG7	E1-like activating enzyme involved in the 2 ubiquitin-like systems required for cytoplasm to vacuole transport (Cvt) and autophagy. Activates ATG12 for its conjugation with ATG5 as well as the ATG8 family proteins for their conjugation with phosphatidylethanolamine. Both systems are needed for the ATG8 association to Cvt vesicles and autophagosomes membranes. Required for autophagic death induced by caspase-8 inhibition. Required for mitophagy which contributes to regulate mitochondrial quantity and quality by eliminating the mitochondria to a basal level to fulfill cellular energy requirements and preventing excess ROS production	**5.877**	0.525	0.732
	*GABARAP*	Gamma-aminobutyric acid receptor-associated protein	Ubiquitin-like modifier that plays a role in intracellular transport of GABA(A) receptors and its interaction with the cytoskeleton. Involved in apoptosis. Involved in autophagy	1.688	0.574	**2.196**
	*MAP1LC3A*	Microtubule-associated protein 1A/1B light chain 3A	Ubiquitin-like modifier involved in formation of autophagosomal vacuoles (autophagosomes)	**0.352**	0.927	**0.119**
	*MTOR*	Serine/threonine-protein kinase mTOR	Serine/threonine protein kinase which is a central regulator of cellular metabolism, growth and survival in response to hormones, growth factors, nutrients, energy and stress signals. MTOR directly or indirectly regulates the phosphorylation of at least 800 proteins. Functions as part of 2 structurally and functionally distinct signaling complexes mTORC1 and mTORC2 (mTOR complex 1 and 2). mTORC1 also negatively regulates autophagy through phosphorylation of ULK1	0.700	0.914	0.779

a The description of gene function has been taken from OMIM^®^ database (accessed in July 2016, www.omim.org).

## Footnotes

1 Similar information, but with less details, was provided as Supplementary material for the paper "Nucleo-mitochondrial interaction in yeast response to cadmium sulfide quantum dots exposure" [29]. This is because the same batch of CdS QDs was used as the one used here.
